# Encouraging adolescents to contact their GP: a community-based trial

**DOI:** 10.3399/bjgp14X679688

**Published:** 2014-04-28

**Authors:** Svein Aarseth, Ingvild Dalen, Ole Rikard Haavet

**Affiliations:** Department of General Practice, Institute of Health and Society, University of Oslo, Norway.; Department of General Practice, Institute of Health and Society, University of Oslo, Norway.; Department of General Practice, Institute of Health and Society, University of Oslo, Norway.

**Keywords:** adolescent behaviour, adolescent health services, confidentiality, general practice, healthcare-seeking behaviour, health education

## Abstract

**Background:**

Adolescents, especially males, often fail to see their GP.

**Aim:**

To determine whether an informative letter could enhance the accessibility and utilisation of healthcare facilities and services.

**Design and setting:**

A community-based trial in one town in Oslo, using a retrospective control group.

**Method:**

GPs in one town in Oslo sent a personal, informative letter at the beginning of 2008 and 2009 to individuals in their practice population who were turning 16 years of age that year. The pooled data for the same year for each surgery were collected. Retrospective data from 1990 and 1991 served as controls for the intervention groups of data collected in 2006 and 2007 respectively. An International Classification of Primary Care-2 diagnosis was given for each contact.

**Results:**

The proportion of adolescents in contact with a GP increased from 59% in the control group to 69% in the intervention group (*P*<0.001). For the males, the increase was from 54% to 72% (*P*<0.001). This reduced sex differences in healthcare seeking. For diagnoses mentioned in the informative letter the incidence rose from 38% in the control group to 55% in the intervention group (*P*<0.001). For the females, there was a non-significant increase in the proportion in contact with the GP, from 63% to 66% in control and intervention groups, respectively. The most frequent contact reasons were respiratory disorders, followed by general and unspecified complaints, skin disorders, musculoskeletal disorders, and psychological disorders. This pattern did not change because of the intervention.

**Conclusion:**

An information letter about health problems and health rights (such as the protection of the adolescent’s privacy) seems to enhance the accessibility and utilisation of GPs, as measured by contact rate, particularly for males.

## INTRODUCTION

Adolescents experience barriers and have unmet needs in the primary healthcare setting.^1^,^2^ A UK report from 2010 stated:

*‘The general practice has a particularly important role, as the hub of a network of services, to ensure that the services are sustainable over time.’*^3^

Adolescents who fail to see a GP do so for several reasons, including embarrassment, lack of trust in protection of privacy in the GP’s surgery, and the belief that their health issue is unimportant.^4^,^5^ They may have poor experience of care,^6^ or the care given might have been unsatisfactory.^3^,^7^ The young people’s needs include issues relating to contraception, menstruation, acne, illness in the family, and arguments with parents.^8^ In general, although many young people report their physical problems to the health services, a large percentage of adolescents with mental health issues do not seek help.^9^ However, adolescents with psychological problems do see their GP for other reasons prior to unveiling the psychological problem.^9^ Females are the most frequent users of youth health clinics and school-based health care in Norway and in other countries.^10^

Young males, especially those who are economically and socially marginalised, seem not to utilise healthcare services.^7^ In Norway, for example, only 10% of adolescents contacting GPs are males,^11^ and males, in general, utilise health services to a lesser extent than females do.^12^ Probable reasons for both sexes are lack of information about primary health care, lack of understanding of their own problems, and concern about or discomfort with GPs’ communication skills.^13^,^14^

The health services for adolescents in Norway include the GP in a regular-scheme general physician system (where all Norwegian citizens are entitled to have a personal regular GP), and low-threshold services, such as drop-in youth health clinics, school health services, telephone support, and clinics for sexual transmitted diseases. Until the age of 16 years, GP consultation is free of charge; after that a small fee is paid up to an annual maximum for GP visits and medication.

Newborns are put on the same GP list as their mothers and, therefore, most adolescents in Norway have the same GP as their mothers until they are 16 years old. According to Norwegian health regulations, adolescents have the right to choose their own GP from the age of 16 years, at which time they also attain their full right to privacy.

Deane *et al* found that information about practical issues concerning consulting healthcare professionals increased intention to seek help.^15^ Adolescents themselves report that they prefer to seek help from professionals that they know and trust.^16^

The purpose of this study was primarily to determine if an informative, personal letter to 16-year-olds from their GP, addressing health issues of importance to adolescents, would enhance the accessibility and attractiveness of healthcare facilities and services, as measured by increased healthcare utilisation. A secondary goal was to obtain information about the reasons for their healthcare seeking, as diagnosed by the GP.

**How this fits in**

Adolescents with health questions may hesitate to contact their GP. Embarrassment, mistrust of their GP’s ability to maintain confidentiality, and the belief that their problems are of minor importance are three major barriers young people report. An information letter about these issues and about health rights, such as the protection of the adolescent’s privacy, enhanced the utilisation and attractiveness of healthcare facilities and services, as measured by the contact rate for young males.

## METHOD

### Study population and participants

In this study of 16-year-old adolescents, all 34 GPs in 12 surgeries in one town in Oslo, were invited to participate; 12 of these GPs were female and 22 were male.

### Measurements

The intervention constituted the mailing of an informative letter at the beginning of 2008 and 2009 to adolescents born in 1992 and 1993, respectively. No personally identifiable information was collected. The dataset for each entry included year of birth, sex, ICPC-2 diagnosis,^17^,^18^ and contact number in the current year. Data were retrieved from the surgeries at the beginning of 2010. Those born in 1990 and 1991 served as historical controls and their data were collected for 2006 and 2007 respectively. The sex of the letter recipient was not registered, but sex is equally distributed in junior high schools and in the directory of residents for age groups in question in the studied town, allowing the study to make an accurate assumption about the sex distribution of the letter recipients.

The letter was composed on the basis of issues expected to be of importance to 16-year-olds, and a focus group with teenagers was used to target the message. The information in the letter addressed the obligation of the GP and the practice staff to maintain confidentiality, the right to choose a GP, the opinion that nothing was too insignificant to discuss, treatment and referral, how to make contact, payment regulations, and examples of problems and diseases that may be of concern to adolescents (Box 1).

**Box 1. Contents of the informative letter sent to adolescents**

**Examples of what you may discuss with your GP**

- Allergy: diagnosis and treatment
- Generally: health concerns
- Headache: right use of medication
- Skin: acne and eczema
- Sports-related injuries
- Sexually transmitted diseases: chlamydia, herpes, genital warts
- Contraception, sex and life together
- Psychological: anxiety, depression, eating disorders etc
- Sick leave

The GP assigned one or more ICPC-2 diagnoses for each consultation, which were used to make an estimate and/or assessment of the reasons for their healthcare seeking. The data comprise the first diagnosis in the registration year.

### Statistics

SPSS (version 8) was used for statistical analysis, unless otherwise stated. The results are given as counts, proportions, and rates. Confidence intervals (CIs) for proportions and for differences between proportions were estimated using online calculators. The χ^2^ test was used to compare groups with regard to proportions of adolescents seeing a GP; both overall and stratified by sex. CIs for the mean rates were based on the normal approximation. These results were also corroborated by bootstrap percentile intervals based on 2000 re-samplings.

## RESULTS

Out of a total of 34 GPs, 33 participated in 2008 and 32 in 2009. Data were collected from their surgery as if they had participated, however, and results are thus given as ‘intention to treat’. There were 975 16-year-olds in the intervention group and 978 in the control group. As shown in Table 1, there was a statistically significant increase in GP utilisation overall after the intervention: the proportion of adolescents in contact with a GP increased from 59% in the control group to 69% in the intervention group (*P*<0.001). For males, contact proportion increased from 54% to 72% (*P*<0.001) and proportion of contact for diagnoses mentioned in the letter increased from 38% to 55% for the control and intervention groups, respectively (*P*<0.001). The increase for females, from 63% (control group) to 66% (intervention group), was not significant (*P* = 0.3). The average number of contacts per patient was the same in the control and intervention groups (Table 1), with females having more contacts than males in both groups.

**Table 1.** GP utilisation by adolescents overall and after intervention

	**Control group**			**Intervention group**		
	
	**Males (*n* = 489)**	**Females (*n* = 489)**	**Total (*n* = 978)**	**Males (*n* = 487)**	**Females (*n* = 488)**	**Total (*n* = 975)**
Proportion seeing GP, ratio (95% CI)	0.54 (0.50 to 0.59)	0.63 (0.58 to 0.67)	0.59 (0.55 to 0.62)	0.72 (0.68 to 0.76)^a^	0.66 (0.62 to 0.70)	0.69 (0.66 to 0.72)^a^
Proportion seeing GP for reason mentioned in letter,^b^ ratio (95% CI)	0.38 (0.29 to 0.47)	0.50 (0.41 to 0.59)	0.44 (0.38 to 0.50)	0.55 (0.46 to 0.64)	0.52 (0.43 to 0.61)	0.54 (0.48 to 0.60)
Total number of contacts	709	1043	1752	966	1126	2092
Contacts per person in group under study, ratio (95% CI)	1.5 (1.3 to 1.6)	2.1 (1.9 to 2.4)	1.8 (1.6 to 1.9)	2.0 (1.8 to 2.2)	2.3 (2.1 to 2.6)	2.2 (2.0 to 2.3)
Contacts per patient attending the surgery, ratio (95% CI)	2.7 (2.4 to 2.9)	3.4 (3.1 to 3.7)	3.0 (2.8 to 3.3)	2.8 (2.5 to 3.0)	3.5 (3.2 to 3.8)	3.1 (2.9 to 3.3)

a P<0.001 for difference between proportions in intervention and control groups.

b As diagnosed by the GP and relating to first contacts only.

**Figure 1.**
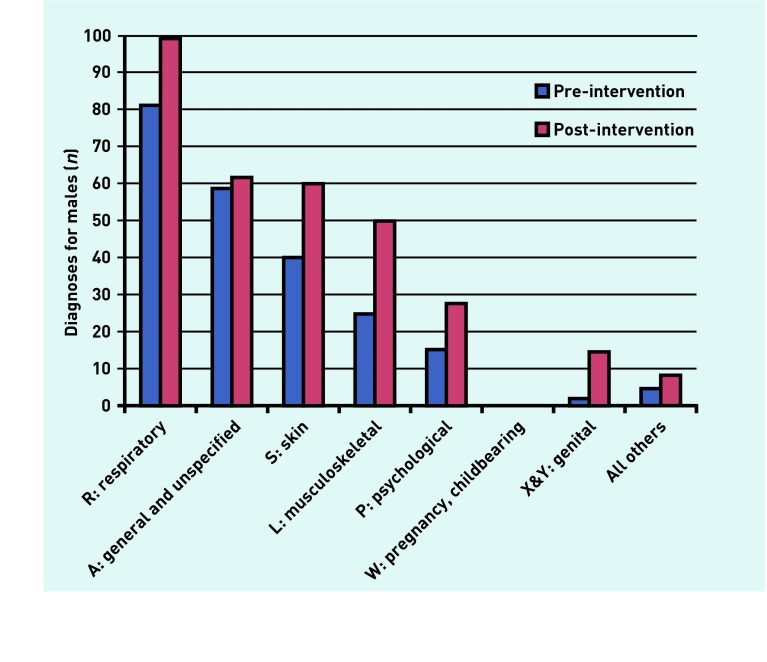
***Number of ICPC-2 diagnoses for males on first contact, by diagnosis chapter in pre-intervention (n = 225) and in post-intervention group (n = 315).***

**Figure 2.**
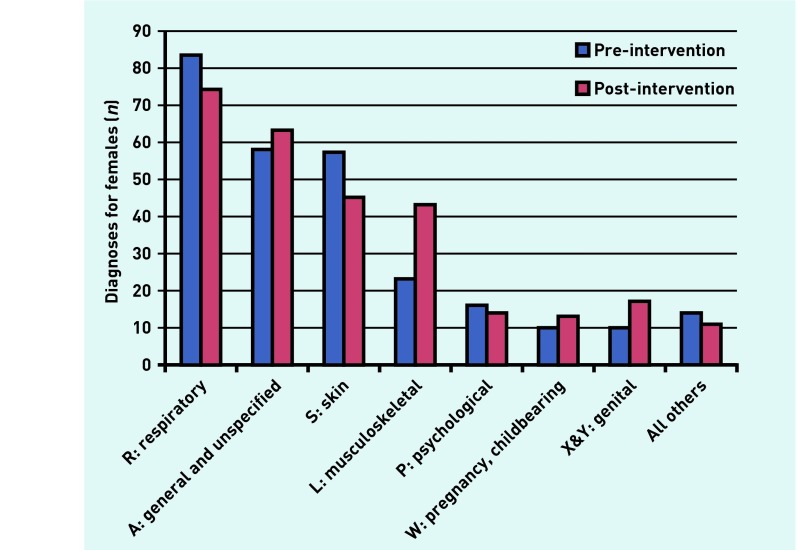
***Number of ICPC-2 diagnoses for females on first contact, by diagnosis chapter in pre-intervention (n = 271) and in post-intervention group (n = 280).***

The distribution of diagnoses related to the first contact for the control and the intervention groups are outlined in Figure 1 and Figure 2 for males and females respectively. The disorders (ICPC-2 Diagnosis Chapters R, A, S, L and P; see Figures 1 and 2), constituted 80% of the contacts (psychological disorders alone comprised 6%).

The most frequent contact reasons were respiratory disorders, followed by general and unspecific complaints, skin disorders, musculoskeletal disorders, and psychological disorders. The intervention did not alter this pattern.

Diagnoses mentioned in the letter were significantly more frequent in the intervention group overall, an effect caused by the behaviour of intervention group males.

## DISCUSSION

### Summary

An informative letter to 16-year-old adolescents in a town in Oslo promotes increased use of primary healthcare services for the overall group and in particular for males. As far as the study is aware this is a novel finding, which may be a low cost, feasible approach for increasing GP-seeking behaviour among adolescents. Among females there was a minor, non-significant increase in healthcare seeking. In addition, females initially have a higher, and perhaps more adequate use of GPs, as they are more likely to attend when they have a problem. The Committee on Adolescents from the American Academy of Paediatrics^19^ states that an adolescence healthcare system should have seven characteristics: availability, visibility, quality, confidentiality, affordability, flexibility, and coordination. The Norwegian regular-scheme GP system meets these demands, except for affordability, as patients of ≥16 years of age must pay a minimal fee for services. The International Covenant on Economic, Social and Cultural rights also states that information is a crucial accessibility issue.^20^ The informative letter was meant as an information tool.

To the best of the study’s knowledge, there are few interventions designed to increase GP contacts among adolescents, but an Australian study has found an increase in intention to contact the GP.^15^

### Strength and limitations

Norwegian health authorities have been intent on developing a low threshold for use of health services among adolescents, some of which are severely under-utilised particularly by males. The intervention in this study has been shown to help ensure that this objective can be reached.

The study chose written, personal information to enhance the knowledge of health services and also provided the GP’s name, address and other contact information, enabling the adolescent to establish contact easily. Problems and issues were identified by literature studies and the chosen topics were approved by the adolescents in the focus group. The focus group had a strong influence on the wording in the letter and the study believes that this specific information led to the increase in the use of the GPs; yet it is a weakness in the study that these individual factors were not analysed. The average number of contacts per patient, nonetheless, was similar in the control and intervention groups. Using a focus group with teenagers to direct the message in the information letter increased the validity. The reliability of the study is strong, since pooled data from each GP practice was used. There are no lost records and the population studied was considered to represent the average, or slightly above the average, of socioeconomic differences in Oslo and Norway.

Yet there are some limitations to be considered; because pooled data from the clinics were collected, there is no information on the sex choice of GP among the males and females. In particular, it is not known whether the non-compliance of the two GPs may have influenced the results more than others. Newborns are put on the same GP list as their mothers and as female patients are more likely to choose female GPs, most adolescents are familiar with their mother’s doctor, so it would be expected that more female doctors saw the group under study. Data for sex were not collected in the population under study, however, data from school population, and directory of residents established that there are equal numbers of males and females in the study town, and it has been assumed that the study population is comparable. None of the doctors had specific training in ‘friendly caring’ for adolescents, but two doctors also served in a youth health clinic. When this study was performed, e-mail, SMS were not used as communication tools in the participating surgeries.

In every municipality, public policies recommend drop-in youth health clinics and school-based health care. In Oslo, where this study was conducted, there is also a clinic for sexually-transmitted diseases and hotlines for support and advice. No reliable information was available on the frequency of use of these services, and it may be seen as a limitation of the study that these factors were not taken into account.

The fact that a larger proportion of adolescents attended the surgeries may be interpreted as implying that the intervention changed their GP-seeking. However, the number of contacts per patient was unchanged indicating that care given to each patient did not changed.

This study represents an intervention to improve the contact between adolescents and GPs. GPs have a legal obligation to their populations, and because they represent continuity, they can serve as an interested adult and health carer. Health facilities and healthcare utilisation may both be viewed as protecting factors, according to Blum *et al*.^21^ An information letter to 16-year-olds appears to be one measure for encouraging adolescents to utilise healthcare services.

This current study compared the intervention group to a historical group. The limitations of an uncontrolled study are acknowledged and although historical data can be confounding, as they do have the potential to give misleading results, these data were from the same GP practices, with the same GPs, and there were no other factors known, such as serious societal events, that would provide confounding issues. The groups are also socioeconomically equal, and comprise students from the same schools.

### Comparison with existing literature

This study shows the proportion of patients that contact their GP and number of contacts per year was in accordance with other studies. In this study 54% of the males in the control group and 72% in the intervention group saw a GP during the study year. The corresponding numbers for females were 63% and 66%, respectively. Kramer *et al*^22^ found that 54% of the adolescents attended the practice in one year. Haavet *et al*^18^ found similar results in their 2001 Oslo study: 59% of the males and 66% of the females, none of whom had undergone intervention, contacted a GP over the course of the year. In their study there were 1.8 contacts per male and 2.1 per female per year. Correspondingly, Hetlevik *et al*^23^ found 1.4 contacts per year for males and females combined. The diagnoses were also grouped in the same manner as Hetlevik *et al* did.

The town that served as the site of this study also runs a youth health clinic, which, unfortunately, does not report its activities. Haavet *et al*^11^ found that patients using this resource also see their GP. It is known that only 5–8% of the contacts in youth health clinics are males.^17^,^22^ A drop-in clinic for sexual and reproductive health issues (‘Sex and Society’) reports that 22% of its contacts in 2009 were male.^24^ This study’s authors agree with Gleeson *et al*^1^ that diversity may be good as long as it does not lead to fragmentation and competition. There must be a hub where information is coordinated, which is responsible for a sustainable service for adolescents, and in Norway, this should be the GP.^3^

There is information on why young people consult the GP, however as health services organisation differs, comparison between countries may be difficult.^9^,^12^,^25^,^26^ A Norwegian study^2^ found that 6.9% of adolescents had sought help during a 12-month period for mental health issues; this corresponds with the current finding of 5.9%. Another important health issue in adolescents is asthma; Furu *et al*^27^, who estimated asthma prevalence by using prescription data, found that 5% of 16-year-old males and a greater proportion of females have asthma. Yet only 2.7% of participants in the current study sought support from their GP for asthma. This finding may indicate that asthma is usually treated by physicians other than GPs (such as paediatricians). Sexual health matters, such as sexually transmitted diseases and contraception, are to a great extend solved in the low-threshold services.

### Implication for research and practice

This study suggests that a personal letter with information to adolescents can facilitate contact with their GP, particularly for males. It is a tool that every GP could start using as it is easily administrated from the practice, and should of course also lead to a youth-friendly practice profile. The intervention should be supported by system training for the whole practice to ensure that young people (especially young males) are well received when they do book GP appointments and attend the practice. More research should be done on what information is crucial and how it alters the adolescent’s healthcare seeking pattern. Cooperation between health services in the area as well as adolescents preferences on communication methods will be of interest in future studies. This study was performed in a regular scheme GP system and may be applied whenever the surgery has a defined population responsibility. It is also believed that the information given in the letter may be used in a broader context, for instance in schools.

## Open access

This article is Open Access: CC BY NC 3.0 license (http://creativecommons.org/licenses/by-nc/3.0/).
